# Small Marine Protected Areas in Fiji Provide Refuge for Reef Fish Assemblages, Feeding Groups, and Corals

**DOI:** 10.1371/journal.pone.0170638

**Published:** 2017-01-25

**Authors:** Roberta M. Bonaldo, Mathias M. Pires, Paulo Roberto Guimarães, Andrew S. Hoey, Mark E. Hay

**Affiliations:** 1 Departamento de Ecologia, Instituto de Biociências, Universidade de São Paulo, São Paulo, São Paulo, Brazil; 2 School of Biology and the Aquatic Chemical Ecology Center, Georgia Institute of Technology, Atlanta, Georgia, United States of America; 3 Australian Research Council Centre of Excellence for Coral Reef Studies, James Cook University, Townsville, Queensland, Australia; Leibniz Center for Tropical Marine Ecology, GERMANY

## Abstract

The establishment of no-take marine protected areas (MPAs) on coral reefs is a common management strategy for conserving the diversity, abundance, and biomass of reef organisms. Generally, well-managed and enforced MPAs can increase or maintain the diversity and function of the enclosed coral reef, with some of the benefits extending to adjacent non-protected reefs. A fundamental question in coral reef conservation is whether these benefits arise within small MPAs (<1 km^2^), because larval input of reef organisms is largely decoupled from local adult reproduction. We examined the structure of fish assemblages, composition of fish feeding groups, benthic cover, and key ecosystem processes (grazing, macroalgal browsing, and coral replenishment) in three small (0.5–0.8 km^2^) no-take MPAs and adjacent areas where fisheries are allowed (non-MPAs) on coral reefs in Fiji. The MPAs exhibited greater species richness, density, and biomass of fishes than non-MPAs. Furthermore, MPAs contained a greater abundance and biomass of grazing herbivores and piscivores as well as a greater abundance of cleaners than fished areas. We also found differences in fish associations when foraging, with feeding groups being generally more diverse and having greater biomass within MPAs than adjacent non-MPAs. Grazing by parrotfishes was 3–6 times greater, and macroalgal browsing was 3–5 times greater in MPAs than in non-MPAs. On average, MPAs had 260–280% as much coral cover and only 5–25% as much macroalgal cover as their paired non-MPA sites. Finally, two of the three MPAs had three-fold more coral recruits than adjacent non-MPAs. The results of this study indicate that small MPAs benefit not only populations of reef fishes, but also enhance ecosystem processes that are critical to reef resilience within the MPAs.

## Introduction

In recent decades, fish biomass and coral cover on many tropical reefs have been severely depleted [1–4]. While this degradation may be associated with numerous factors, overfishing has been a primary driver of declines in fish biomass [1, 3, 4]. In many regions, the removal of large herbivorous fishes has been linked to changes in the benthic condition of coral reefs and the replacement of corals by benthic algae (and sometimes other non-scleractinian coral organisms) [3, 5, 6]. Indeed, a number of studies report positive correlations between live coral cover and the biomass of herbivorous fishes [2, 7, 8], so reduced biomass of herbivorous fishes may be linked to lower resilience of coral reef ecosystems, as coral health is compromised by competitive interactions with seaweeds that escape regulation by herbivory [2, 3, 9].

The establishment of no-take marine protected areas (MPAs) is a common management strategy to conserve healthy coral reefs and enhance the recovery of degraded systems [10–14]. No-take MPAs are commonly applied as part of community-based and/or government-driven management schemes to sustain and enhance coral reefs and their associated fisheries [15–17]. Large MPAs and networks of large MPAs are acknowledged for their efficiency in protecting fish and coral assemblages, as well as ecosystem processes, on tropical reefs [e.g., 14, 18, 19]. Conversely, the results of studies about the effectiveness of small MPAs (here considered a MPA with a total area < 1 km^2^) are inconsistent, despite the existence of many small reserves throughout the South Pacific [e.g., 15] and other regions [e.g., 8, 12]. Indeed, a recent review indicates that 60% of the existing marine no-take areas are smaller than 1 km^2^ [20]. The role of closure size in marine reserve design is important because small MPAs may not enclose the entire home range or fulfill the habitat requirements of target species and lead to a decoupling of local larval production and recruitment [16, 21–25]. As a consequence, recovery of fish assemblages, and of the ecological processes linked to these species, could be compromised in small reserves.

The role of MPAs in the recovery of degraded coral reefs is usually assessed through changes in state variables such as fish biomass and coral cover; few studies have quantified the effect of MPAs on ecological processes and social interactions. Very little is known, for example, on how important ecosystem processes, such as herbivory and the replenishment of coral populations, are impacted by the implementation of MPAs. Additionally, although many reef fish species frequently feed in groups [26–30], no studies, to our knowledge, have investigated the consequences of ecosystem protection on the structure of fish feeding groups in marine ecosystems. Regardless of the causes of group formation, the composition of feeding groups in terrestrial systems has been shown to change under different levels of predation pressure [26, 31, 32] and human disturbance [33]. Given that fish behavior [34, 35] and the composition of fish assemblages [2, 18, 36] are known to differ between reefs under different fishing pressure, the structure of fish groups may also be influenced by the implementation of MPAs. Hence, further information on the role of small reserves in the recovery of state variables, ecological processes and social interactions in degraded coral reefs is key to a better understanding of the potential benefits of this strategy.

To understand the effect of small MPAs on both state variables and processes, we compared three small MPAs and adjacent areas where fishing is allowed (non-MPAs) in Fiji. More specifically, we addressed the five following questions: (1) Do MPAs have greater diversity, density, or biomass of fishes than non-MPAs?, (2) Are fish feeding groups larger, more diverse, and composed of larger individuals?, (3) Are rates of grazing and macroalgal browsing higher inside MPAs?, (4) Do MPAs have higher coral cover and lower macroalgal cover than non-MPAs?, (5) Do MPAs have higher densities of coral recruits compared to adjacent non-MPAs? If MPAs are effective in preserving the health of coral reefs, we should expect greater coral cover, diversity, biomass and density of fishes and fish groups, as well as greater herbivory rates and density of coral recruits inside these areas.

## Materials and Methods

The study was conducted from November 2010 through February 2011 and between November 2011 and January 2012 on shallow (~1 m below the surface at low tide, equal or shallower than 2 m at high tide), intertidal fringing reefs platforms (up to 800-m wide) along the Coral Coast (18° 13.05’S, 177° 42.97’E) of Viti Levu, Fiji’s main island. Many of the owners of traditional fishing rights along the Coral Coast have established small, customary no-take MPAs to improve and sustain their adjacent fishing grounds. The MPAs in this region are delimited by surface markings and enforced by local villagers, and they have been closed to all fishing activities since their inception (about 10 years). The only exception to this closure was a small experimental hook and line fishing research project that was conducted in the MPAs of Votua and Namada [see 37 for details]. In the non-MPAs, the main fishing targets are species of Acanthuridae (Nasinae), Epinephelidae, Labridae, Mullidae, and Lutjanidae (RMB and MEH, pers. obs.). Permission for the research was granted by the Fijian Ministry of Education, National Heritage, Culture & Arts, Youth & Sports, which is authorized to approve field studies in Fijian waters. No animal collection or experimental procedures involving animals were conducted during the study, and no endangered species were recorded during our assessments. RMB conducted all of the visual surveys described below, and RMB and ASH conducted the algal assays.

**Fig 1:**
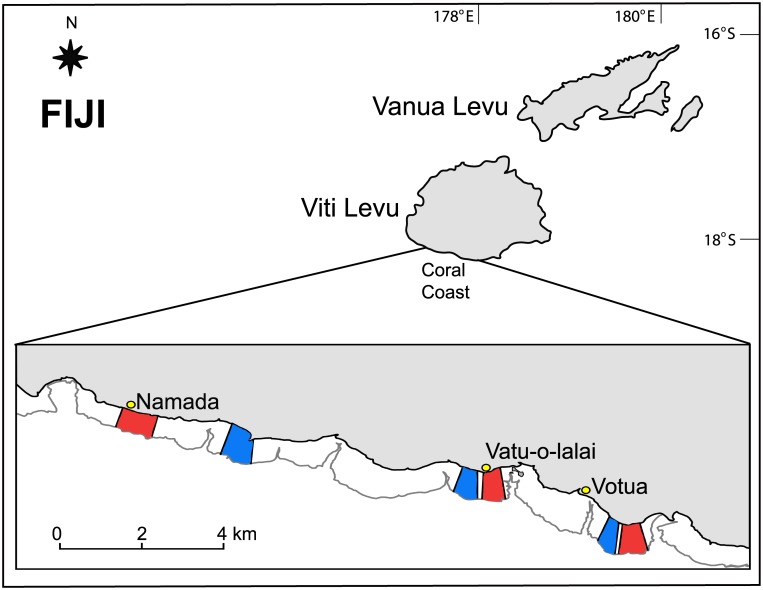
Study sites. Marine protected areas (red) and adjacent areas, where fisheries are allowed (green), at three village sites (Votua, Vatu-o-lalai, Namada) along Fiji’s Coral Coast.

To assess the effects of MPAs on fish assemblages, fish feeding group composition, herbivory rates, benthic cover, and coral recruit density, we compared three spatially paired MPA and adjacent, fished, areas (non-MPAs) associated with the villages of Votua, Vatu-o-lalai and Namada (Fig 1). Comparisons of fish assemblages inside and outside of closures are widely used for determining the effects of reserves [11, 37, 38], but it should be acknowledged that this approach does not reveal the state of an MPA relative to an undisturbed baseline. Unfortunately, such undisturbed baselines rarely exist [39] and are difficult to reconstruct [40, 41], so long-term, strict before-after-control-impact (BACI) experimental designs are uncommon for evaluating coral reef management [but see 13, 42]. Given the urgent challenge of understanding how coral reefs are affected by human impact, less robust methods must be employed to evaluate and inform management impacts [13, 43].

The studied MPAs were established in 2002 (Vatu-o-lalai, Namada) and 2003 (Votua), and shortly after establishment, coral cover was low (~7%), and macroalgal cover was high (~35–45%) in both the MPAs and non-MPAs [2]. Each MPA covers an area of less than 1 km^2^, and paired MPAs and non-MPAs have similar depth and current regimes. All surveys and algal assays were performed toward the center of each MPA and approximately 300–600 m from the adjacent non-MPA survey site (i.e., approx. 150–300 m from either side of the MPA boundary). All surveys and assays were conducted during the same season (austral summer) to minimize seasonal variation in sampling. The reef extends approx. 1 km from shore within each MPA and non-MPA, and all data were collected between 30 and 700 m of the shore (i.e., shoreward of the reef crest) parallel to the shoreline [44].

### Fish assemblages

Underwater visual censuses (UVC; [13, 18, 45]) were used to assess fish assemblages in MPAs and non-MPAs at the three village sites. Underwater visibility at all study sites (> 15 m) was appropriate for the use of UVC, but due to the visual limitations of this method, we did not consider cryptic species or species with a maximum total length < 5 cm. During our surveys, we categorized species into two major categories (Herbivores and Non-herbivores) that were subdivided into ten sub-categories (S1 Table, S1 Fig) [46, 47]. Herbivores include the main roving nominally herbivorous fish clades, which play an important role in the control of benthic algae [3, 5], and these species were further divided into four sub-categories (browsers, grazers, scrapers, and excavators) according to diet, feeding mode, and impact on the benthos [47–49]. Some sub-categories within Herbivores were also based on taxonomic groups because some feeding modes are exclusive to certain fish taxa, so our designations were as follows: (1) browsers remove mature fleshy macroalgae; (2) grazers typically crop algal turfs, leaving the basal portions of the algae intact (includes the detritivorous *Ctenochaetus striatus*); (3) scraping parrotfishes feed predominantly on algal turfs by scraping the reef matrix; and (4) excavating parrotfishes feed on algal turfs, but also remove pieces of the underlying substratum when feeding [48, 49] (S1 Table).

The category of Non-herbivores includes all species that feed on other, non-algal resources, and these fishes were classified into six sub-categories based on the primary prey [46]: (1) corallivores mostly feed on scleractinian corals; (2) cleaners predominantly feed on ectoparasites on other reef species; (3) mobile invertebrate feeders consume mobile invertebrates and small fishes; (4) sessile invertebrate feeders predominantly consume sessile invertebrate species other than scleractinian corals; (5) omnivores are generalist species that feed on a variety of algal and animal items; and (6) piscivores predominantly feed on fishes (S1 Table).

Separate 30m x 4m belt transects were performed for Herbivores and Non-herbivores. While simultaneously deploying the transect line [following 50], a snorkeler (always RMB) recorded all non-cryptic fishes (either Herbivores or Non-herbivores) within 2 m of either side of the transect. Individual fish were identified to species and placed into 5-cm (total length) size classes, and the lengths were converted to biomass using established length-weight relationships [46]. A total of 186 transects (4 to 6 transects conducted per day) were conducted for Herbivore*s*: 66 in Votua (35 in the MPA and 31 in the non-MPA), 61 in Vatu-o-lailai (30 in the MPA and 31 in the non-MPA), and 59 in Namada (29 in the MPA and 30 in the non-MPA). For Non-herbivores, a total of 183 transects (4 to 6 transects per day): 66 in Votua (35 in the MPA and 31 in the non-MPA), 58 in Vatu-o-lailai (30 in the MPA and 28 in the non-MPA), and 59 in Namada (30 in the MPA and 29 in the non-MPA).

Transects were conducted in each area within 2 h of high tide (approx. 1.5 m depth) and were equally distributed between the two sampling periods (Dec 2010–Jan 2011 and Dec 2011–Jan 2012), the months within each sampled year (December and January of each year). On each sampling day, four to six transects were deployed on the reef parallel to the shoreline, with a minimum of 10m between adjacent transects. To ensure that transects were independent and non-overlapping, small numbered surface floats were placed at the start and end of each transect, and were left in position during all sampling. Care was taken to avoid re-counting fishes that left and subsequently re-entered the transect areas. The initial starting point of the transects for each day was selected based on a map of the study sites with two constraints: (1) as to a minimum distance from shore (at least 30 m), and a minimum distance from the MPA boundaries (150 m). On subsequent days, the snorkeler swam at least 15 m towards the reef crest from the previous transects and started a new set of transects so that different sampling days correspond to different distances to the coastline. Each new set of transects was again selected based on a minimum distance from the previous transects (15 m), and a minimum distance from the MPA boundaries (as above). Hence, at each study site, four to six transects were surveyed per day, with different locations within each site sampled on three non-consecutive days in each year. This procedure provided a comprehensive sampling within each area.

### Fish feeding groups

The structure of fish feeding groups was assessed using a series of 10-min timed transects, which maximized the distance transversed in search of groups rather than being limited to a 30m transect where there may be no groups. A fish feeding group was defined as any aggregation of two or more fish in which individuals were observed feeding or biting a potential food source [following 26]. Pairs of butterflyfishes (f. Chaetodontidae) and leatherjackets (f. Monacanthidae) were not considered to be feeding groups because these species usually live in pairs that are not primarily associated with feeding [46, 51].

A total of 30 timed transects (n = 15 MPA; n = 15 non-MPA) were performed at each of the three village sites (n = 90 transects total) between December 2011 and January 2012. Transects were conducted within 2 h of high tide and equally distributed from 10:00 h–14:00 h, which represents the feeding period for most of the diurnal reef fish species in the study sites [e.g., 46, 52, 53]. A group was counted if at least one individual in the aggregation was inside the transect area. For each feeding group, all individuals were identified to species, their total length (TL) estimated and placed into 5cm size classes. Fish lengths were converted to biomass using established length-weight relationships [46]. For each transect, a snorkeler swam parallel to the reef crest for 10 min at a standard speed and recorded all fish groups within 2 m of each side of the transect. On each sampling day, five transects were deployed on the reef parallel to the shoreline. Adjacent transects were separated by a minimum of 20 m, and small surface floats and reef and shoreline landmarks were used to avoid resampling the same areas. On subsequent sampling days, the snorkeler swam approximately 30 m towards the reef crest and started a new set of transects so that different sampling days correspond to different distances to the coastline. This procedure provided a widespread sampling within each area [46].

### Herbivory rates

Rates of grazing by parrotfishes and macroalgal browsing were assessed across the six study sites using established techniques (e.g., [54–56] for fish grazing; [57–59] for browsing). The feeding rates of parrotfishes were estimated within each of the six study sites from December 2011–January 2012 using remote stationary video cameras; this method was selected as it has been shown to reduce observer effects on fish behavior [57]. Underwater cameras (GoPro) attached to a small lead weight were randomly positioned next to areas covered by algal turfs within each study site, and all feeding on the benthos was recorded for 2 hours. At the start of each video, a length of chain was used to demarcate a 4-m^2^ area and provide a scale for estimating the length of any fishes in the video. The chain was removed after one minute, and the cameras were left to record all feeding activities in the absence of divers. This procedure was repeated in each study site during three periods of the day: morning (07:00–10:00 h), afternoon (12:00–14:00 h) and evening (17:00–19:00 h), resulting in a total of 24 videos (48 h) per site.

To ensure similar sampling effort among sites, sampling was conducted over 18 days, always during high tide. In the first week, during which high tide occurred in the morning, four cameras were distributed in the MPA and four in the non-MPA of a given village, and over the following two days, the same procedure was repeated for the remaining two villages. A few days later, when high tide occurred during the middle of the day, the same procedure was repeated and then repeated again for the afternoon period. This entire sampling scheme was performed twice, so we recorded a total of eight videos per study site per time period within each village. All videos were subsequently viewed, and all parrotfishes observed feeding on the reef substrata were identified to species, and their length estimated. Grazing rates were then calculated as the product of species-specific bite rates and bite areas [following 1, 60], and expressed as the percentage of the 4m^2^ area grazed per day. Species-specific bite areas were obtained from the literature [49], and where these were not available the bite area of a closely related species with a similar feeding type and body size was used.

Macroalgal browsing was assessed at each site using a series of macroalgal assays during December 2011. Five common macroalgal species in the non-MPAs (*Hormophysa triquetra*, *Padina boryana*, *Sargassum polycystum*, *Sargassum* sp., and *Turbinaria ornata*) were collected by hand, spun in a salad spinner for 20 revolutions to remove water and weighed [following 61]. One thallus of each alga was randomly selected and attached at equal intervals along a 60-cm length of 3-ply rope by inserting the holdfast between the strands [following 62]. The order of the algal species along the rope was randomized among replicates. Three replicate assays (or ropes) were exposed to herbivores, and three assays were placed in exclusion cages (60 x 20 x 20 cm, 1-cm square mesh) at each site and left on the reef for 5 h. Assays within each site were separated by 20–50 m. After 5h the assays were collected and each thallus was carefully removed from the rope, spun and weighed (as described above), and the reduction in algal biomass was calculated.

### Benthic cover

The benthic cover of the six study sites was surveyed along 30-m long transects running parallel to the shore in each MPA and non-MPA. Along each transect, photographs were taken from 0.5 m above the bottom every 2 m along each transect (i.e., 16 photos per transect), so that consecutive photographs did not overlap. The area of each photograph was about 25 cm x 30 cm, therefore sampled area per transect was about 1.2m^2^. Photos were analyzed for percentage cover of corals and macrophytes using CPC with Excel extensions [63]; the program randomly placed 20 points on each photo, and we identified the organism beneath each point. Organisms were further classified into four main categories: scleractinian corals, macroalgae, epilithic algal matrix [the EAM sensu 64], and others.

A total of 273 transects were conducted: 87 in Votua (48 in the MPA and 39 in the non-MPA), 94 in Vatu-o-lalai (44 in the MPA and 50 in the non-MPA), and 87 in Namada (41 in the MPA and 51 in the non-MPA). Transects were conducted from December 2010 to January 2011 and from December 2011 to January 2012, and there was no spatial overlap between transects even between different sampled year periods. On each sampling day, four to six transects were deployed on the reef parallel to the shoreline. To ensure that transects were independent and non-overlapping, they were separated by a minimum of 15 m, and the ends of each transect were marked with small surface buoys. Additionally, after conducting every 4–6 transects, the snorkeler swam approx. 25 m towards the reef crest to start a new set of transects, so sampling was widespread within each area.

### Density of coral recruits

To assess the effect of the MPAs on the replenishment of coral populations, the density of coral recruits on natural reef substrata was assessed at night during January and February 2011 using a fluorescence technique [65]. A coral recruit was defined as a post-settlement coral ≤ 5 mm in its longest dimension [following 51]. When excited with blue light, both corals and their symbiotic dinoflagellates autofluoresce, so we conducted counts with a prototype lighting system with a yellow filter attached to a diving mask (Night Sea) [65] at night, when autofluorescence could be detected and recruits would be most evident.

Coral recruit counts were conducted along a series of 50-m long transects positioned parallel to shore, and a total of 18 transects (n = 9 MPA; n = 9 non-MPA) were performed at each of the three village sites (n = 54 transects total). Ten 25 cm x 30 cm rectangular quadrats were randomly placed along each transect with a minimum distance of 2 m between quadrats (540 plots total). Once a quadrat was deployed on the substratum, all coral recruits within the borders of the quadrat were counted. Plots were not placed on sand or surfaces densely covered by macroalgae because initial trials using this technique revealed that these substrata were totally devoid of coral recruits (RMB pers. obs.).

### Statistical analyses

We used Generalized linear mixed models (GLMM) implemented under a Bayesian framework to test the effect of protection status (MPA vs. non-MPA) on the abundance and biomass of each subcategory of Herbivores and Non-herbivores at the three village sites. We used the same approach to test the effect of protection status on the number of individuals, biomass and diversity of species in the observed feeding groups. The models have a hierarchical structure where the protection status is nested within site. For abundance and biomass of Herbivores and Non-herbivores, we used muti-response models where each subcategory is a separate response variable [66]. Because we have several samples for the same site, transect and sampling day were included as random factors. For the richness and abundance model, we used a Poisson error structure given the nature of the data. For biomass and diversity data we used a Gaussian error structure. We performed separate analyses for Herbivores and Non-herbivores. We compared model fit against a benchmark model in which protection status was not included as a fixed effect using the Deviance information criterion (DIC; [67]). The MCMC used to sample the posterior distributions of effect sizes ran for 10^6^ iterations and was sampled every 100 iterations (thinning = 100) after burn-in (5×10^5^). We considered effect size significant when the 95% credible interval of the estimated posterior distributions of parameters did not include 0. We monitored chain mixing by checking the effective sample sizes (ESS) for fixed and random effects. We used inverse gamma priors for variance components [66]. Exploratory analyses indicate that estimates for fixed effects were robust to prior selection. Outliers were removed prior to the GLMM analyses to reduce overdispersion, although analyses with and without the outliers yielded qualitatively similar results. We used the R [68] package MCMCGLMM [66] for all analyses based on GLMMs.

We compared benthic cover between MPAs and non-MPAs using three-way ANOVA, with village site (Votua, Vatu-o-lalai, and Namada), status (MPA and non-MPA) and year (2010/2011 and 2011/2012) as fixed factors. Separate ANOVAs were used to compare the percentage cover of four different substratum types (scleractinian corals, macroalgae, epilithic algal turfs and others). Benthic cover data were arcsine-transformed, and fish density and biomass data were log-transformed to meet assumptions of normality (frequency histograms). When differences were significant, the test was followed by specific planned comparisons between paired treatments (MPA vs non-MPA) at each village site. P-values were adjusted with the Holm-Sídák method, in which the adjusted p-value is equal to 1 − (1 − *unadjusted Pvalue*)^*k*^, where k refers to the number of comparisons.

The rates of grazing and browsing and the density of coral recruits were compared between MPAs and adjacent non-MPAs using two-way ANOVA with status (MPA and non-MPA) and village site (Votua, Vatu-o-lalai, and Namada) as fixed factors. Separate analyses were used to compare (1) parrotfish grazing rates, (2) macroalgal browsing rates, and (3) the number of coral recruits per quadrat (log-transformed). Holm-Sídák-adjusted paired comparisons were also used when differences were significant. ANOVAs for benthic cover, grazing and browsing rates, and density of coral recruits, as well as all graph plots in this manuscript, were programmed in R 3.0.1 using base package functions [68].

## Results

### Fish assemblages

**Fig 2:**
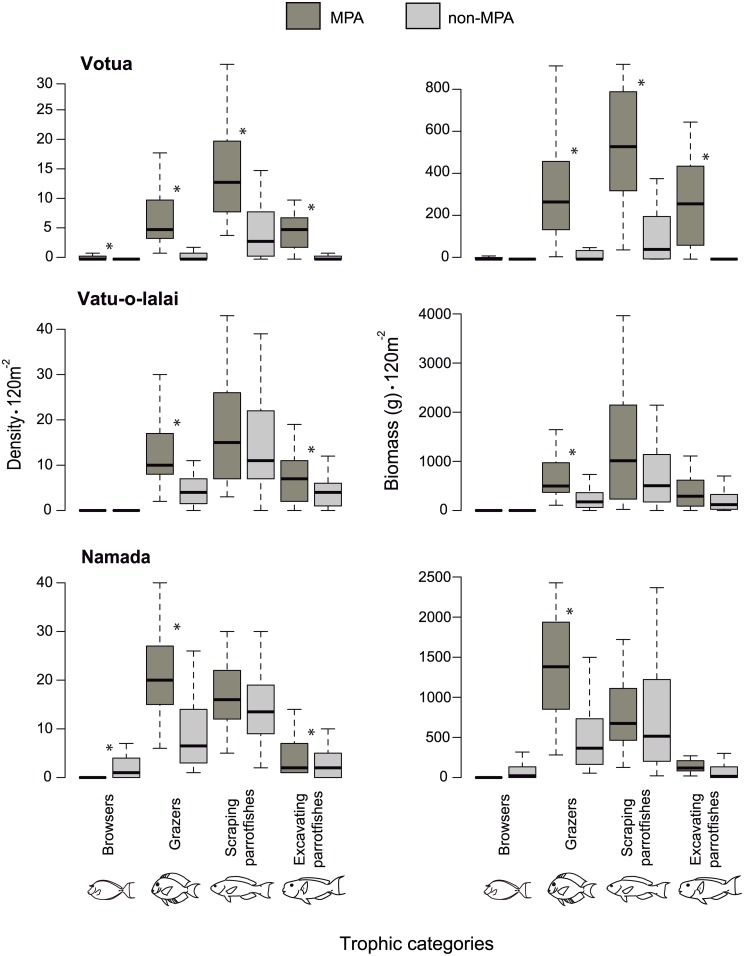
Herbivorous fishes. Box plot for fish density and biomass (120 m^-2^) of four categories of herbivorous fishes (browsers, grazers, excavating parrotfishes, and scraping parrotfishes) in MPAs and adjacent non-MPAs at three village sites (Votua, Vatu-o-lalai, and Namada) along the Coral Coast of Fiji. * signals the comparisons in which the 95% credible interval indicates a significant effect of protection status.

**Fig 3:**
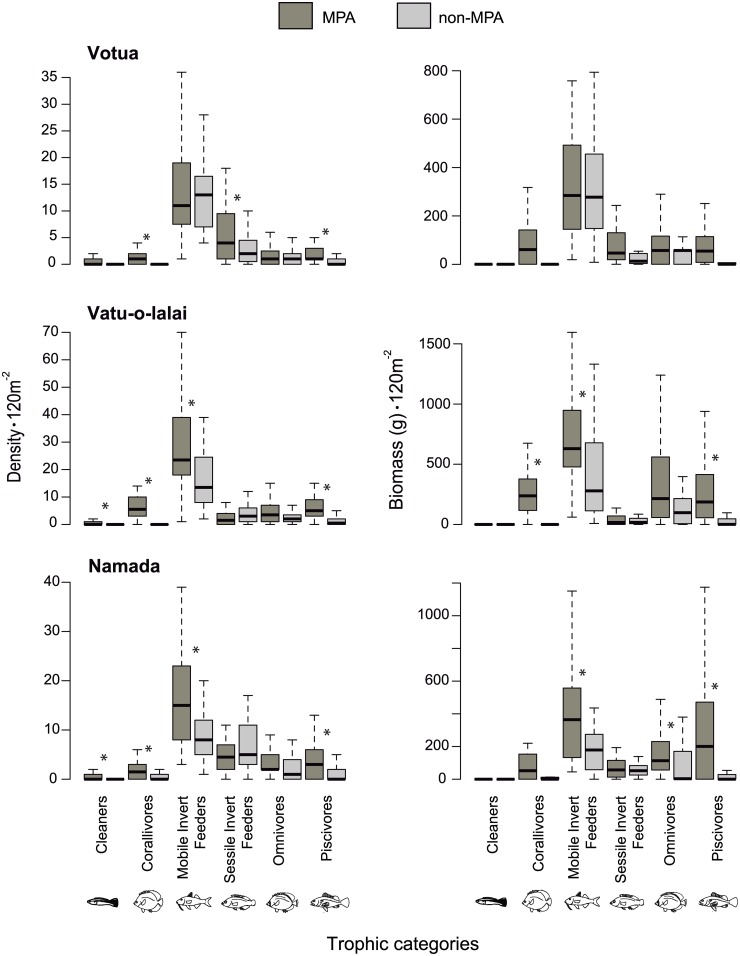
Non-herbivorous fishes. Density and biomass (120 m^-2^) of five categories of Non-herbivores. Study sites and symbols as in Fig 2.

Protection status affected richness, abundance and biomass of most feeding subcategories of Herbivores (Fig 2) and Non-herbivores (Fig 3) within each site. The models including protection status were always favored when compared to models including site and trophic categories as fixed effects, but without protection status (see DIC values in Table 1). Including year as a random effect did not improve model fit and including day as a random factor only improved fit in a few models (Table 1).

**Table 1:** Deviance information criterion (DIC) for models including (Full model) or not (Reduced) protections status as a predictor variable. Smaller DIC signal preferred models (in bold).

Response variable	Full model	Reduced
Herbivores		
Richness	**680.67**	762.11
Abundance	**2932.24**	3001.75
Biomass	**9792.83**	9860.19
Non-herbivores		
Richness	**857.79**	918.38
Abundance	**3702.79**	6155.60
Biomass	**12269.83**	12328.82
Feeding groups		
Number of individuals	**4208.16**	4214.48
Diversity of species	**682.55**	710.42
Biomass	**8922.30**	9008.10

The richness of herbivores and non-herbivores was higher in MPAs across all three village sites (Table 2). Indeed, the number of herbivorous species per transect was, on average, 1.4 to 3.3 times larger in MPAs than in adjacent non-MPAs. For non-herbivorous species, MPAs contained 1.5 to 1.6 times more species than non-MPAs (Table 2).

**Table 2:** Model results for the observed richness of herbivores and non-herbivores. Parameter estimates (posterior mean), with 95% credible interval (CI) and effective sample size (ESS), for each level and interactions between levels of fixed factors (and variance associated with random factors). Effect sizes of the interaction site:status are relative to benchmark levels (non-MPAs to MPAs of each site). Text in bold highlight the effects deemed significant according to the 95% CI.

Effect	Estimate	95% CI		ESS
Herbivores (Site: status)				
Votua	**-1.19**	**-1.52**	**-0.87**	**290.26**
Vatu-o-lalai	**-0.52**	**-0.76**	**-0.29**	**578.87**
Namada	**-0.38**	**-0.63**	**-0.15**	**590.5**
Random				
Votua: transects	0.003	0	0.008	2498.724
Vatu-o-lalai: transects	0.003	0	0.007	2053.906
Namada: transects	0.003	0	0.007	2704.427
Residual	0.003	0	0.009	1207.778
Non-herbivores				
Votua	**-0.43**	**-0.63**	**-0.24**	**1150.38**
Vatu-o-lalai	**-0.5**	**-0.67**	**-0.33**	**1454.36**
Namada	**-0.4**	**-0.59**	**-0.2**	**1188.45**
Random	0.007	0	0.023	1244.976
Votua: transects	0.004	0	0.011	1491.386
Vatu-o-lalai: transects	0.003	0	0.009	2458.816
Namada: transects	0.003	0	0.007	3614.512
Residual	2.49	2.37	2.6	2051.66

The effects of MPAs on the fish density and biomass of the subcategories of Herbivores was not uniform across all sites. The MPAs at some villages had greater densities and biomasses of grazers, scraping parrotfishes, and excavating parrotfishes than non-MPAs. The abundance and biomass of grazers was smaller in non-MPAs when compared to MPAs at the three studied villages (95% credible intervals—CI density: Votua: [-2.53, -1.43]; Vatu-o-lalai: [-1.48, 0.6]; Namada: [1.37, -0.49]; biomass: Votua: [-439.82, -96.79]; Vatu-lalai: [-568.51, -209.52]; Namada: [-714.37, -311.2]). Other herbivore categories had more variable responses, with lower abundance or biomass in certain sites but not in others according to protection status (Fig 2, S2 Table). For instance, density of excavating parrotfishes was higher in the MPA than in the non-MPAs at all three village sites (Votua: [-2.76, -1.49]; Vatu-o-lalai: [-1.17, -0.15]; Namada: [-0.97, -0.03]), but differences in biomass between the MPA and non-MPA were only detected at Votua (Votua: [-437.75, -86.03]; Vatu-o-lalai: [-322.31, 54.69]; Namada: [-257.55, 109.86]). Density of browsers was higher inside than outside the MPA in Votua (CI = [-2.84, -0.08]), but similar between the MPA and the non-MPA in Vatu-o-lalai (CI = [-0.52, 1.89]; Fig 2, S2 Table). At Namada, density of browsers was higher in the non-MPA than in the MPA (CI = [0.42, 2.98]), which was primarily due to the large numbers of small rabbitfish, *Siganus spinus*. In contrast, browser density at the Namada MPA was dominated by the unicornfish *Naso unicornis*, which was absent in the non-MPA despite the relatively short distance between the two areas.

Among Non-herbivores, although the overall trend also showed a reduction in abundance and biomass in non-MPAs in comparison to paired MPAs, subcategories also varied in their response to protection among sites (Fig 3, S3 Table). For instance, density of corallivores was higher within the MPA than in the non-MPA of the three village sites (Votua [-2.42, -0.76]; Vatu-o-lalai [-2.99, -1.44]; Namada [-2.38, -0.83]), but biomass only differed between the MPA and non-MPA of Vatu-o-lalai (Votua [-106.80, -15.57]; Vatu-o-lalai [-108.19, -33.10]; Namada [-111.91, -15.56]). Mobile invertebrate feeders had higher density and biomass inside than outside the MPA at Vatu-o-lalai (density: [-1.01, -0.16]; biomass: [-227.27, -91.76]) and Namada ([-1.01, -0.19]; [-238.66, -107.16]), but not differences for these comparisons were found at Votua ([-0.35, 0.43], [-4.32, 115.64]. Piscivores had higher density in MPAs than in non-MPAs at all three village sites (Votua [-1.97, -0.63]; Vatu-o-lalai [-2.07, -0.91]; Namada [-1.77, -0.56]). Biomass of this group was also higher within than outside MPAs of Vatu-o-lalai (CI = [-176.74, -40.07]) and Namada (CI = [-146.43, -7.61]), with no significant MPA vs. non-MPA differences at Votua (CI = [-100.77, 20.54]).

### Fish feeding groups

A total of 853 fish groups were recorded from timed transects, with 503 groups in the MPAs and 350 groups in the non-MPAs (Votua: 175 vs 132; Vatu-o-lalai: 178 vs 106; Namada: 150 vs 112). The total species richness of fish groups, after pooling transect data within each study site, was greater within the MPAs than the non-MPAs (25 vs 15 at Votua, 28 vs 22 at Vatu-o-lalai, and 27 vs 16 at Namada). The fish species in feeding groups within MPAs vs non-MPAs respectively comprised 60% vs 55% of species recorded at Votua, 68% and 62% at Vatu-o-lalai, and 61% and 50% at Namada. Therefore, the relative number of shoaling species was higher inside the MPAs. In all areas, more than 80% of fish groups were dominated by nominally herbivorous species, such as the parrotfishes *Chlorurus spilurus* and *Scarus psitttacus*, and the surgeonfishes *Acanthurus triostegus*, *Ctenochaetus striatus* and *Zebrasoma velifer*. Small invertebrate feeders, such as *Halichoeres trimaculatus* and *Thalassoma hardwicke*, also frequently occurred in groups, especially in mixed shoals with herbivores.

Differences between the fish groups in MPAs and non-MPAs were detected for fish biomass at all three village sites. The biomass of the fish groups was, on average, 2.6, 1.3, and 2.6 fold greater in MPAs than in non-MPAs in Votua (93.7 ± 6.7 and 32.3 ± 3.2 kg per group, respectively; 95% CI = [-71.36, -31.52]), Vatu-o-lalai (145.8 ± 16.4 and 387.8 ± 25.1; [-73.61, -36.07]) and Namada (249.9 ± 30.4 and 58.7 ± 9.4; [-85.35, -44.30]). The species diversity of fish groups in MPAs was higher than in non-MPAs in Votua (0.34 ± 0.04 and 0.13 ± 0.02 species per group, [-0.3, -0.13]) and Vatu-o-lalai (0.40 ± 0.03 and 0.27 ± 0.03; [-0.18, -0.02]) but not in Namada (0.27 ± 0.03 and 0.21 ± 0.02; [-0.15, 0.02]).

The mean number of individuals per fish group did not differ between MPAs and non-MPAs within each village (Votua: [-0.15, 0.33]; Vatu-o-lalai [-0.14, 0.22]; Namada: [-0.26, 0.15]). However, fish groups of more than 61 individuals were only observed within MPAs: five each in Votua and Vatu-o-lalai and eight in Namada (S2 Fig). In all areas, fish groups of more than 50 individuals were composed almost exclusively, and in some cases exclusively, by the grazer *Acanthurus triostegus*.

### Herbivory rates

**Fig 4:**
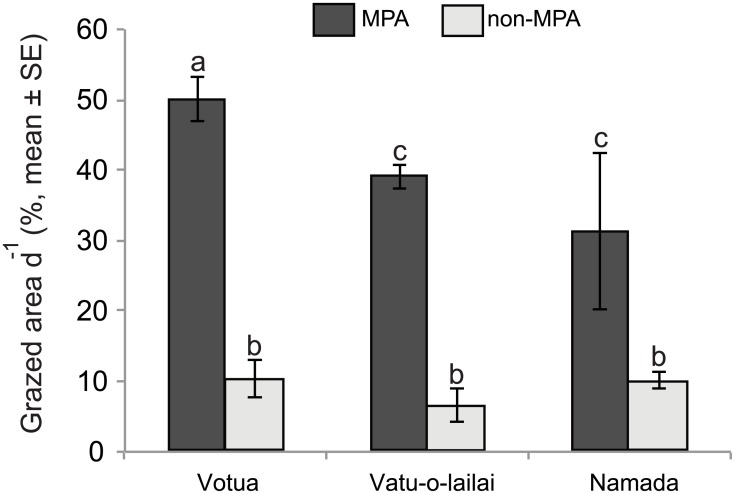
Grazing by parrotfishes. Rates of substratum grazing by parrotfishes (% grazed area d^-1^, mean ± SE) in MPAs and adjacent non-MPAs at three village sites (Votua, Vatu-o-lalai, and Namada) along the Coral Coast of Fiji. * and ** indicate paired bars with significant (p < 0.05) or highly significant (p < 0.001) differences, respectively. Note the different scales of the y-axes.

**Fig 5:**
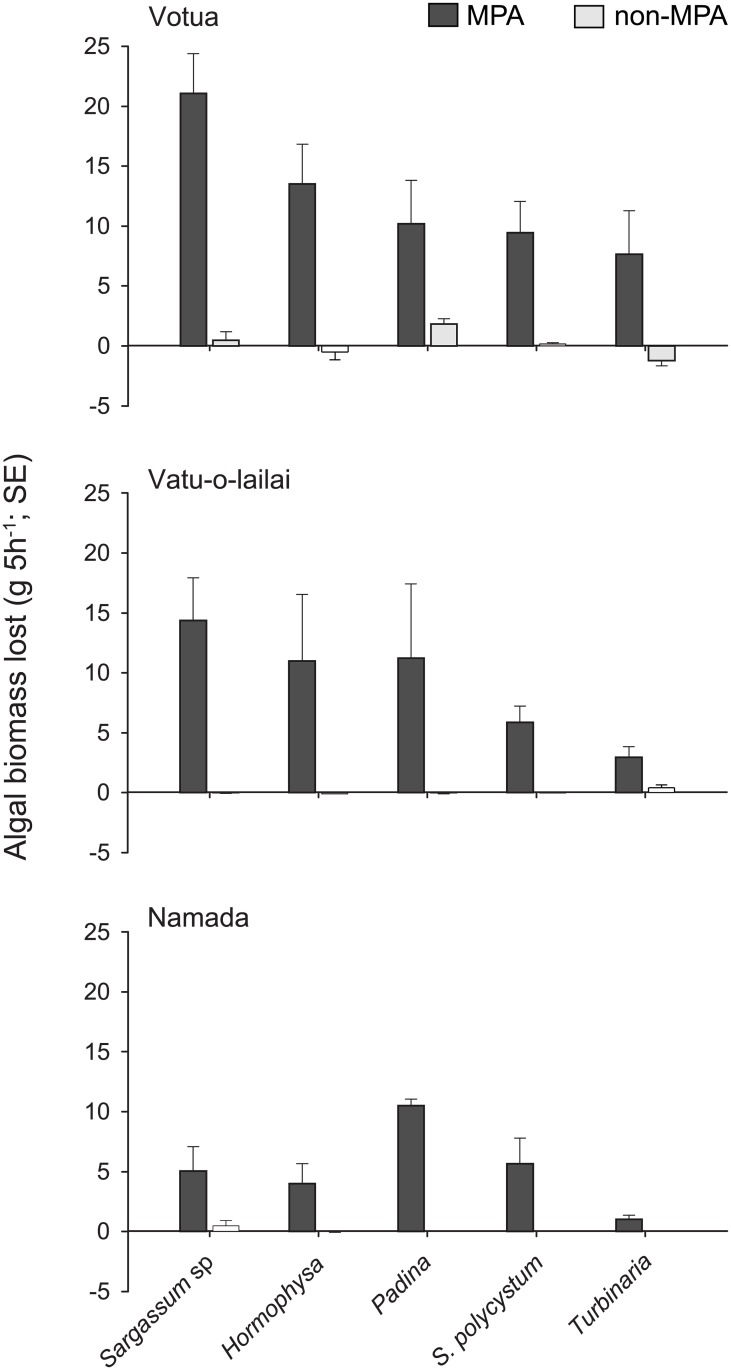
Macroalgal browsing. Rates of macroalgal removal by herbivores (% of algae consumed in 5 h, mean ± SE). Study sites and symbols as in Fig 2.

MPAs had higher estimated rates of parrotfish grazing and macroalgal browsing than adjacent non-MPAs in the three village sites (Figs 4 and 5, respectively). Differences in parrotfish grazing were significant between MPAs and non-MPAs (Status: F = 191.11, *df* = 1, p < 0.001) and among village sites (Site: F_2_ = 6.78, p = 0.003), with Votua presenting significantly higher values than the other two sites. Parrotfish grazing (% grazed area of plot d^-1^, mean ± SE) was, respectively, 4.8, 5.9 and 3.1 times higher in MPAs vs non-MPAs at Votua (50.09 ± 3.13 vs 10.40 ± 2.65; t = 3.28, *df* = 48, p = 0.006), Vatu-o-lalai (39.15 ± 1.76 vs. 6.58 ± 2.35; t = 3.28, *df* = 48, p = 0.006), and Namada (31.31 ± 4.17 vs 10.09 ± 1.20; t = 3.60, *df* = 48, p = 0.002; Fig 4).

There were large and consistent differences between the MPAs and non-MPAs in the consumption of the five brown macroalgae species (Fig 5), but no site effect was detected (Site: F ≤ 3.67, *df* = 2, p ≥ 0.06 for the five species). The reduction in the biomass of all five macroalgae exposed to herbivores was significantly greater in MPAs than in non-MPAs (Status: F ≥ 10.86, *df* = 2, p ≤ 0.001 for all species tested), ranging from 81.5–86.6% 5 h^-1^ for *Padina* to 13.9–57.4% 5 h^-1^ for *Turbinaria* (Fig 5). In contrast, the reduction in algal biomass in the non-MPAs was less than 4.0% 5 h^-1^ for all macroalgae except *Padina* within the Votua non-MPA (14.4% 5 h^-1^). Reductions in algal biomass within the exclusion cages were negligible across all sites (< 2.6% 5 h^-1^).

### Benthic cover

**Fig 6:**
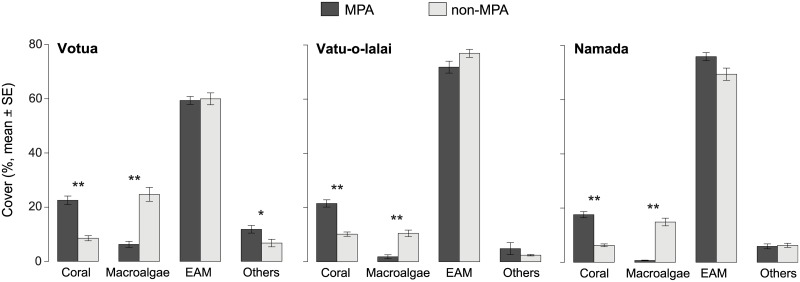
Benthic cover. Percentage cover (mean ± SE) of four categories of benthos (scleractinian corals, macroalgae, eplithic algal matrix and others) in MPAs and adjacent non-MPAs, at three village sites (Votua, Vatu-o-lalai, Namada) along Fiji’s Coral Coast. * and ** indicate, respectively, paired bars that differ significantly (p < 0.05) and highly significantly (p < 0.001).

MPAs at Votua, Vatu-o-lalai, and Namada had, on average, 260%, 210%, and 280% as much coral cover, respectively, and only 5%, 17%, and 25% (Status: F = 182.22, *df* = 1, p < 0.001) as much macroalgal cover as their paired non-MPAs (F_1_ = 24.42, p < 0.001; Fig 6, S5 and S6 Tables). Coral cover (mean ± SE) ranged from 17.6 ± 1.6% to 22.5 ± 1.1% inside the MPAs vs 6.3 ± 0.5% to 10.2 ± 0.8% in the non-MPAs (p < 0.001 for all MPA vs non-MPA comparisons within villages, S6 Table). Similarly, macroalgal cover ranged from 0.7 ± 0.1% to 6.3 ± 1.1% in the MPAs vs 10.5 ± 1.2% to 24.7 ± 2.6% in the non-MPAs (p < 0.001 for all MPA vs non-MPA comparisons within each village, S6 Table). Cover of the epilithic algal matrix, the dominant benthic component across the six study sites, and others was similar across this six study sites, except for the cover by others in Votua, which was significantly higher within the MPA (t = 2.95, *df* = 261, p = 0.009, Fig 2). No effect of year was detected for any of the benthic cover comparisons (S5 Table).

### Coral recruits

**Fig 7:**
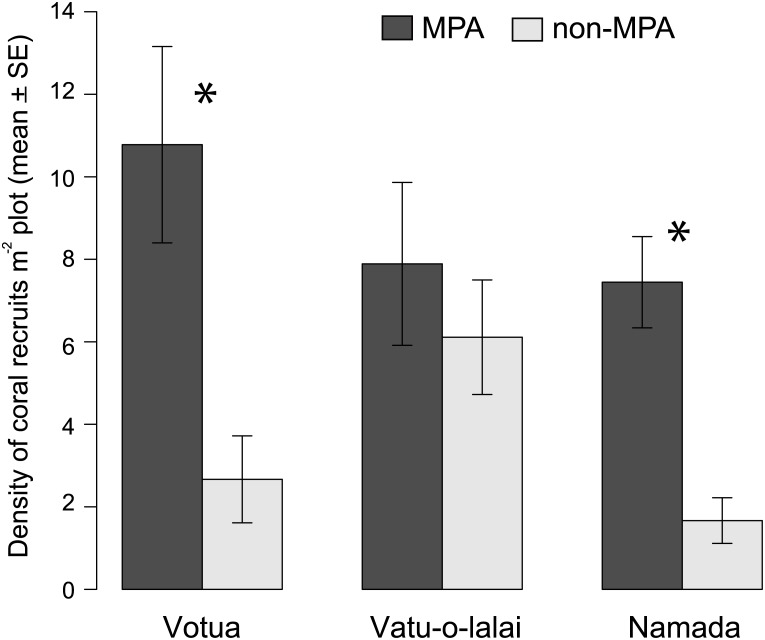
Coral recruits. Density (mean ± SE) of coral recruits m^-2^ of plot area (see text for details). Study sites and symbols as in Fig 2.

The density of coral recruits differed between MPAs and non-MPAs (Status: F = 18.27, *df* = 1, p < 0.001) but not among village sites (Site: F = 1.47, *df* = 2, p = 0.24). The density (mean ± SE per m^-2^) of coral recruits on natural reef substrata was approximately three times higher in MPAs vs non-MPAs at Votua (10.8 ± 2.4 vs. 2.7 ± 1.0; t = 3.28, *df* = 48, p = 0.006) and Namada (7.4 ± 1.1 vs. 1.7 ± 0.5; t = 3.6, *df* = 48, p = 0.002; Fig 7). At Vatu-o-lalai, the density of recruits did not differ significantly between the MPA (7.9 ± 2) and the non-MPA (6.1 ± 1.4; t = 0.52, *df* = 48, p = 0.94; Fig 7). Interestingly, the density of coral recruits in the non-MPA at Vatu-o-lalai was significantly higher than in the other two non-MPAs and did not differ from the MPAs at Votua and Namada. The density of coral recruits was similar between areas with the same status in Votua and Namada (Fig 7).

## Discussion

MPAs are widely used for conserving fish populations and coral cover and, in doing so, it is hoped they will improve the recovery of reefs after disturbances [8, 11, 12, 69]. In this study, protection from fishing within small MPAs not only increased the species richness, density, and biomass of fishes, but also increased key ecosystem processes (herbivory), the diversity of fish groups, the coral cover, and the density of coral recruits. Indeed, despite the small size of the MPAs in this study, we recorded a 3–6 fold higher grazing by parrotfishes and a 3–5 fold higher macroalgal browsing in MPAs compared to adjacent non-MPAs, and this was associated with 2.6–2.8 greater coral cover within the MPAs. Collectively, these findings demonstrate that even small (< 1 km^2^) and young (ca. 10 years) MPAs can effectively conserve fish communities [e.g., 70, 71, 72], with significant effects on fish group formation, herbivory rates, and coral cover [e.g., 8, 73]. Also, as young corals were more abundant within than outside two out of three MPAs, it is possible that these small MPAs can provide higher abundance of coral recruits on the reef.

Herbivorous fishes are widely recognized as being critical to the resilience of coral reefs, but these species are heavily targeted by fishers in many regions [1, 5, 9]. In the present study, protection from fishing led to higher species richness of herbivorous fishes, specifically higher densities and biomasses of grazing surgeonfishes and rabbitfishes, within all three MPAs versus adjacent non-MPAs. Indeed, previous studies indicate that species in these clades have high site fidelity and home ranges of less than 100,000 m^2^ [e.g., 74, 75, 76], which could be totally encompassed by the studied MPAs (about 500,000–800,000 m^2^ each). However, the abundance of scraping parrotfishes was not affected by protection within two of the three MPAs, and the biomass of macroalgal browsing fishes were either equal to or lower within MPAs than adjacent non-MPAs.

Parrotfishes and the dominant browsing fishes in the studied reefs (i.e., *Naso unicornis* and *N*. *lituratus* [59]) are targeted by spearfishers in the non-MPAs at the study sites (RMB, ASH, and MEH pers. obs.) and thus may be expected to respond strongly to protection. In the case of browsers, it is possible that the higher abundance of feeding resources (i.e., macroalgal cover) in the non-MPAs influenced populations of these species, driving them out of the MPAs. However, the same would not be applicable to grazers, including the parrotfishes, given the similar abundance of EAM inside and outside the MPAs. Still, despite the apparent lack of a consistent numerical response in abundance and biomass, herbivory by browsers and grazers was markedly greater within the MPAs than the non-MPAs, and this inconsistency between abundance (or biomass) estimates and rates of herbivory is likely, at least in part, related to the behavioral responses of fishes to fishing and to the presence of the diver conducting the fish counts. Numerous studies have shown that browsing fishes are often underrepresented in visual surveys due to their wary nature [57, 61, 77]. Furthermore, reef fishes, and parrotfishes in particular, have been shown to be warier of divers in areas where they are targeted by spearfishers [78, 79]. Given the small size of the MPAs in the current study and the MPA study sites being within 300–600 m of the reserve boundary, resident fishes may have been wary of divers both within non-MPAs and the adjacent MPAs. This increased wariness may have contributed to the discrepancies between the visual assessments of abundance and the estimated rates of grazing and browsing in the absence of divers. Further studies on fish wariness on the studied MPAs and non-MPAs would be necessary to assess the role of this factor in the assessment of local fish assemblages.

Alternatively, the higher rates of macroalgal browsing and grazing within the MPAs may be related to differences in the availability of algae between the MPAs and non-MPAs. For example, the reduced browsing rates estimated using assays within the non-MPAs could be a consequence of the dilution of browsing across the higher abundance of macroalgae at these sites as has been recorded elsewhere [80, 81]. However, if this was the case for the higher browsing rates within the MPAs, the higher algal biomass in non-MPAs could be a result of two processes: (1) less grazing in the MPA (i.e. lower rates of macroalgal loss) and (2) greater productivity in the non-MPA (i.e., greater rates of algal biomass production). Previous studies in these locations demonstrated that there were no differences in rates of macroalgal settlement or growth between MPA and non-MPA locations [82], that nitrogen availability did not differ between MPA and non-MPA sites [83], and that macroalgae grew as well or better in the MPA than the non-MPA sites when protected from consumers [84], probably due to the decreased competition among macroalgae within the MPA sites where macroalgae were rare. Thus, considerable evidence suggests faster macroalgal removal by herbivores in the MPAs, with no evidence for more rapid growth of macroalgae in the non-MPAs. Although we cannot discard the possibility that some of the lower grazing rate measures in the non-MPAs are due to the herbivore/algal mass ratio, the available information suggests that algal accumulation in the non-MPAs is due primarily to low removal rates.

While the influence of macroalgal biomass on our estimated rates of browsing cannot be discounted, the availability of the EAM, the preferred feeding substratum of grazing fishes, cannot explain the differences in our estimates of grazing between MPAs and non-MPAs. Estimated grazing rates were 3–5 fold greater in MPAs than adjacent non-MPAs, yet the cover of the EAM was broadly comparable between sites. This may be attributed to the higher abundance of some herbivorous fishes within the MPAs, such as scraping parrotfishes in Votua, and excavating parrotfishes and grazers in all three MPAs. Additionally, nutritional quality of the EAM may differ between areas under high and low grazing pressure, as sediment loads are higher in turfs exposed to low grazing activity [85, 86].

The most immediate and apparent effects of reduced herbivory on coral reefs are increases in the cover or standing biomass of algal assemblages [8, 59], however several indirect effects, or feedbacks, manifest through changes in benthic communities [9, 58]. For example, macroalgae can suppress the settlement, growth and survivorship of corals [87–90], thereby limiting the capacity of coral populations to recover following disturbances. Furthermore, areas of high macroalgal biomass have been shown to suppress herbivory [58] leading to a positive feedback that reinforces macroalgal-dominance [58, 83]. The higher coral cover and the lower macroalgal abundance within the three MPAs in the present study, along with the higher density of coral recruits within two of the three MPAs, may be related to the higher rates of herbivory in these areas. Indeed, previous studies at these sites demonstrated that seaweed cover is lower and coral cover is greater in MPAs vs non-MPAs [2, 59], and that macroalgae was rapidly removed when transplanted to the MPAs [59]. In marine reserves in the Bahamas, increased fish grazing as a consequence of reduced fishing has also been correlated with the suppression of macroalgal cover and a two-fold increase in the density of coral recruits [69]. Similarly, on Lord Howe Island, Australia, the density of juvenile corals (< 50 mm diameter) was negatively correlated with macroalgae cover and positively correlated with the biomass of herbivorous fishes [50]. Thus, the reduced fishing and greater herbivory within MPAs may enhance the settlement and survival of juvenile corals by suppressing macroalgal biomass.

In addition to the association of greater herbivory with increased coral abundance in the MPAs versus the non-MPAs, differences in non-herbivorous fish assemblages were also associated with benthic composition of the reefs. Although not measured in this study, benthic carnivores (e.g., corallivores, mobile invertebrate feeders) may have direct effects on the availability of benthic species through consumption [91–93] and indirect effects through feeding or competing with species that interact with benthic species [14, 94, 95]. Therefore, differences in the non-herbivorous fish assemblages, as found in the previous study, also may be linked to some of the contrasts observed in the benthic communities between adjacent MPAs and non-MPAs.

One of the main criticisms of small MPAs is that their areas do not encompass the entire home range of target species [23, 96] and the size of the MPA may not adequately capture ecological processes. However, most reef fish species recorded in our study have relatively small home ranges (less than 100,000 m^2^) and high site fidelity [e.g., 74, 75, 76], so that many individuals probably do not leave the MPA boundaries during their lifetime [74]. Furthermore, a high proportion of fish species (ca 75%) are reliant on live corals at settlement [97, 98] and, as a consequence, reef fish larvae have strong preferences for areas with high coral cover [87]. Indeed, a recent investigation at our study sites found that juveniles of many fish species avoid chemical cues from macroalgae and are attracted to cues from certain corals [51]. The low coral cover and high seaweed cover in the non-MPAs probably suppress the settlement of fishes within the non-MPAs. Thus, the present study indicates that the establishment of small MPAs benefits local reef fish assemblages with potential positive outcomes for fish social interactions, herbivory rates, and the density of coral recruits.

The significant increases in coral cover and in the abundance and biomass of some reef fish categories observed, besides the small size of the reserves, may also been caused, or enhanced, by the fact that MPAs in the studied region are located along the same coast within a relatively short distance (i.e., < 10 km distance between MPAs). Indeed, MPA networks are considered as an effective solution to large-scale reserve coverage as, although restrictions apply to only a small fraction of the exploited area, the presence of multiple source populations could increase the potential benefits to the protected and unprotected areas by increasing the connectivity among them [23, 25, 99]. Therefore, it is probable that the existence of multiple small areas along the Coral Coast explain some of the observed contrasts in benthic cover, fish assemblages, and herbivory rates between the studied MPAs and non-MPAs.

Despite the higher density and biomass of several fish clades within studied MPAs, herbivorous fish assemblages in all six studied reefs were predominantly composed by small and medium sized species (up to 25 cm), such as *Ctenochaetus striatus*, *Chlorurus sordidus*, and *Scarus schlegeli*. No large parrotfishes (e.g., *Bolbometopon muricatum*, *Chlorurus microrhinos*) were detected in our study, even in the grazing assessment videos. Among carnivores, large groupers were rare, the Maori wrasse (*Cheilinis undulatus*) was not recorded in any of our surveys, and only one shark was detected across all transects. This almost complete absence of large species in all our study sites could be a consequence of the small size of the studied MPAs, given that some large fish species usually have larger home ranges [23] that could easily exceed the areas of the studied MPAs [but see 70]. This suggestion is reinforced by the fact that other studies on small and/or young MPAs also found positive increases in fish density of small species, with little enhancement of larger species’ populations, if any [43, 100]. Therefore, although our findings collectively indicate that the small MPAs in Fiji may increase numbers and biomass of fishes, it is possible that the full recovery of these reefs is challenged by the small sizes of these closures, especially for species with large home ranges.

The greater species diversity, maximum size and biomass of fish groups in MPAs versus non-MPAs can cause differences in the impacts of fish feeding. Feeding efficiencies and critical reef processes, such as herbivory and coral replenishment, can be increased by the presence of more diverse fish feeding groups in the MPAs, and such functional effects of consumer diversity have been demonstrated in the few experimental studies conducted to date [e.g., 59, 101]. Moreover, differences in species composition of fish feeding groups may allow for behavioral variations among individuals that forage in groups versus foraging alone or in single-species groups [reviewed by 26, 27]. Similarly, large groups of herbivores, which were only observed within MPAs, may provide access to different food items because some fishes have access to areas held by territorial herbivores when feeding in larger groups [102–104].

Differences in the biomass and diversity of fish feeding groups between the MPAs and non-MPAs could reflect differences in the abundance of shoaling species, predation pressure, or both. However, fish groups were mostly composed of parrotfishes, which were similarly abundant inside and outside MPAs in two out of three village sites, and sessile invertebrate feeders, for which no differences were found between MPAs and non-MPAs in the three study sites. Therefore, differences in fish groups seem to be more related to other contrasts between MPAs and non-MPAs than the abundance of shoaling species. Because protection from predation may be a primary driver of group feeding [29, 105, 106], the lower predation pressure in non-MPAs could reduce the need for group feeding in these areas, although larger fish group sizes may also be a maladaptive in the presence of fishers [107]. Under lower predation pressure, the costs of group feeding, such as competition for resources, may reduce the advantages of this strategy in terms of lower predation risk [26]. In contrast, as MPAs have higher densities and biomasses of piscivores, group formation could represent an important refuge strategy for prey species within these areas [35].

To our knowledge, the present study is the first to establish a link between coral reef protection and group feeding in fishes, and future studies on social feeding should aim to identify the relative roles of protection from predation versus the effects of abundance in driving the differences in fish group formation between MPAs and non-MPAs. As social interactions are linked to population and community dynamics, changes in these interactions can scale up with consequences for the community and ecosystem [108, 109]. For instance, considering that group feeding may influence the amount of time that individuals spend foraging and that group size influences feeding rate, seaweed removal, algal turnover and the recruitment of benthic organisms can all be affected by how individuals aggregate when foraging. Therefore, assessing the effects of reef protection in fish social interactions may provide key information on how MPAs affect the ecosystem as a whole.

In summary, our results indicate that, despite their small sizes, the studied MPA provide increases in fish density and biomass, with benefits extended to fish group formation, herbivory rates, coral cover, and density of juvenile corals. As coral reefs are increasingly degraded by anthropogenic activities, the removal of species and the loss of functional diversity will increase and exacerbate problems linked to overfishing and the loss of species [3, 14]. These problems are often assessed in terms of their direct threats to the dynamics of natural populations (survival and reproduction) and community structure, whereas changes in social and ecological interactions are less frequently addressed, especially for small marine reserves [110, 111]. Assessing different levels of system organization in reefs within versus outside of MPAs can improve our understanding of how habitat degradation affects the ecosystem and will inform the design of more efficient management strategies.

## Supporting Information

S1 Fig Fish species. Examples of fish species in the 10 trophic / functional categories considered in the study. **Herbivores**: (A) browser *Naso unicornis*, (B) grazer *Acanthurus triostegus*, (C) scraping parrotfish *Scarus psittacus*, and (D) excavating parrotfish *Chlorurus spilurus*; **Non-herbivores**: (E) corallivore *Chaetodon trifascialis*, (F) cleaner *Labroides dimidiatus*, (G) mobile invertebrate feeder *Parupeneus bifasciatus*, (H) sessile invertebrate feeder *Halichoeres trimaculatus*, (I) omnivore *Chaetodon ulietensis* and (J) piscivore *Carcharhinus melapterus*. Photos: João Paulo Krajewski.
(TIF)

S2 Fig Fish group size. Boxplots for the number of individual fish (mean ± SE) in feeding groups in marine protected areas (MPAs) and adjacent unprotected areas (non-MPAs) at three village sites (Votua, Vatu-o-lalai, and Namada) along the Coral Coast of Fiji. Each circle corresponds to a fish group. The y-axis is presented in log-scale.
(EPS)

S1 Table Fish categories. Trophic-functional categories considered for the fish species recorded during fish censuses in MPAs and adjacent non-MPAs at three village sites (Votua, Vatu-o-lalai, and Namada) along the Coral Coast of Fiji.
(DOCX)

S2 Table Mixed models herbivorous fish. Results of the models for the observed density and biomass of herbivorous reef fishes (per 120 m^2^). Parameter estimates (posterior mean), with 95% credible interval (CI) and effective sample size (ESS), for each level and interactions between levels of fixed factors (and variance associated with random factors). Effect sizes of the interaction site:status are relative to benchmark levels (non-MPAs to MPAs of each site). Text in bold highlights the effects deemed significant according to the 95% CI.
(DOCX)

S3 Table Mixed models non-herbivorous fish. Results of the models for the observed density and biomass of non-herbivorous reef fishes (per 120 m^2^). Parameter estimates (posterior mean), with 95% credible interval (CI) and effective sample size (ESS), for each level and interactions between levels of fixed factors (and variance associated with random factors). Effect sizes of the interaction site:status are relative to benchmark levels (non-MPAs to MPAs of each site). Text in bold highlights the effects deemed significant according to the 95% CI.
(DOCX)

S4 Table Fish groups. Results of models for the number of individuals, biomass (kg) and diversity of species (Shannon diversity index) of fishes in feeding groups in MPAs and adjacent non-MPAs at three village sites (Votua, Vatu-o-lalai, and Namada) along the Coral Coast of Fiji. Parameter estimates (posterior mean), with 95% credible interval (CI) and effective sample size (ESS), for each level and interactions between levels of fixed factors (and variance associated with random factors). Effect sizes of the interaction site:status are relative to benchmark levels (MPAs of each site). Text in bold highlights the effects deemed significant according to the 95% CI.
(DOCX)

S5 Table Three-way ANOVA of benthic cover. Statistical differences in percent cover (arcsine-transformed data) of scleractinian corals, macroalgae, epilithic algal matrix (EAM) and “others” in MPAs and adjacent non-MPAs at the villages of Votua, Vatu-o-lalai, and Namada along the Coral Coast of Fiji. Significant p-values marked in **bold** (p < 0.05); highly significant p-values (< 0.0001) marked with **.
(DOCX)

S6 Table MPA vs non-MPA benthic cover. Comparison of percentage cover of scleractinian corals and macroalgae in MPAs and adjacent non-MPAs at three village sites (Votua, Vatu-o-lalai, and Namada) along the Coral Coast of Fiji. P-values (Holm-Sídák adjusted; *df* = 261) refer to planned comparisons following ANOVA. Significant p-values marked in **bold** (p < 0.05); highly significant p-values (< 0.0001) marked with **.
(DOCX)
