# Supplementary figures and images for: Small Marine Protected Areas in Fiji Provide Refuge for Reef Fish Assemblages, Feeding Groups, and Corals
Source: PLoS One. 2017 Jan 25;12(1):e0170638. doi: 10.1371/journal.pone.0170638 (PMC5266309; doi:10.1371/journal.pone.0170638)

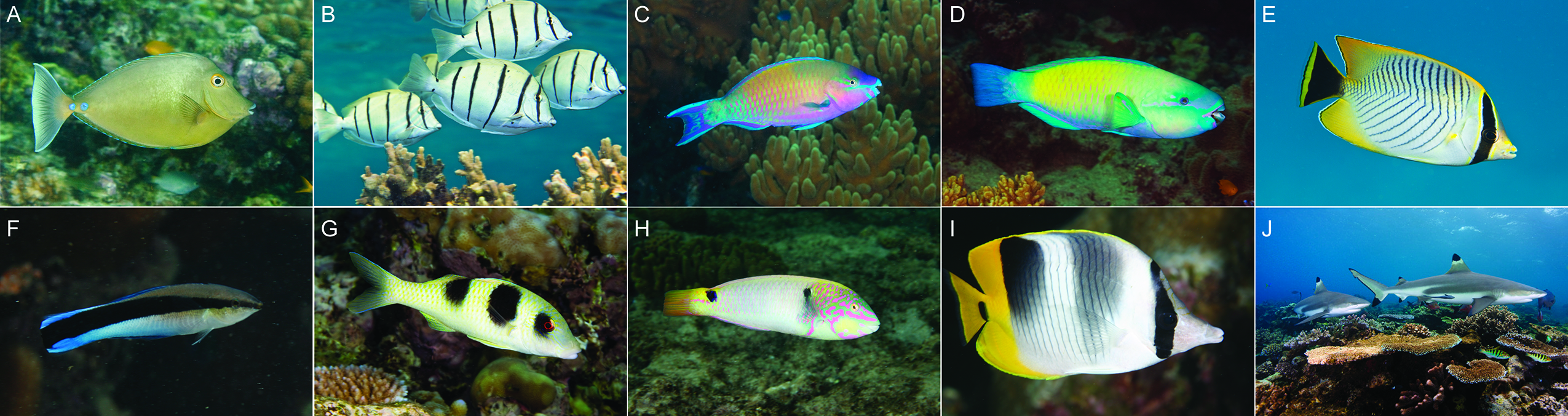

Supplement: S1 Fig — Examples of fish species in the 10 trophic / functional categories considered in the study. Herbivores: (A) browser Naso unicornis, (B) grazer Acanthurus triostegus, (C) scraping parrotfish Scarus psittacus, and (D) excavating parrotfish Chlorurus spilurus; Non-herbivores: (E) corallivore Chaetodon trifascialis, (F) cleaner Labroides dimidiatus, (G) mobile invertebrate feeder Parupeneus bifasciatus, (H) sessile invertebrate feeder Halichoeres trimaculatus, (I) omnivore Chaetodon ulietensis and (J) piscivore Carcharhinus melapterus. Photos: João Paulo Krajewski. (TIF) [file pone.0170638.s001.tif]
