# Supplementary material for: Small Marine Protected Areas in Fiji Provide Refuge for Reef Fish Assemblages, Feeding Groups, and Corals
Source: PLoS One. 2017 Jan 25;12(1):e0170638. doi: 10.1371/journal.pone.0170638 (PMC5266309; doi:10.1371/journal.pone.0170638)
Supplement: S1 Table — Trophic-functional categories considered for the fish species recorded during fish censuses in MPAs and adjacent non-MPAs at three village sites (Votua, Vatu-o-lalai, and Namada) along the Coral Coast of Fiji. (DOCX) [file pone.0170638.s003.docx]

**S1 Table.** **Fish categories.** Trophic-functional categories considered for the fish species recorded during fish censuses in MPAs and adjacent non-MPAs at three village sites (Votua, Vatu-o-lalai, and Namada) along the Coral Coast of Fiji.

| Main  category* | Sub  category** | Family | Species |
| --- | --- | --- | --- |
| H | 1 | Acanthuridae | *Naso lituratus* |
| H | 1 | Acanthuridae | *Naso unicornis* |
| H | 1 | Siganidae | *Siganus spinus* |
| H | 2 | Acanthuridae | *Acanthurus auranticavus* |
| H | 2 | Acanthuridae | *Acanthurus triostegus* |
| H | 2 | Acanthuridae | *Ctenochaetus striatus* |
| H | 2 | Acanthuridae | *Zebrasoma scopas* |
| H | 2 | Acanthuridae | *Zebrasoma veliferum* |
| H | 2 | Siganidae | *Siganus doliatus* |
| H | 3 | Labridae | *Hipposcarus longiceps* |
| H | 3 | Labridae | *Scarus altipinnis* |
| H | 3 | Labridae | *Scarus chameleon* |
| H | 3 | Labridae | *Scarus dimidiatus* |
| H | 3 | Labridae | *Scarus frenatus* |
| H | 3 | Labridae | *Scarus ghobban* |
| H | 3 | Labridae | *Scarus globiceps* |
| H | 3 | Labridae | *Scarus oviceps* |
| H | 3 | Labridae | *Scarus psittacus* |
| H | 3 | Labridae | *Scarus rivulatus* |
| H | 3 | Labridae | *Scarus schlegeli* |
| H | 4 | Labridae | *Chlorurus bleekeri* |
| H | 4 | Labridae | *Chlorurus spilurus* |
| N | 1 | Chaetodontidae | *Chaetodon lunulatus* |
| N | 1 | Chaetodontidae | *Chaetodon melannotus* |
| N | 1 | Chaetodontidae | *Chaetodon ornatissimus* |
| N | 1 | Chaetodontidae | *Chaetodon plebeius* |
| N | 1 | Chaetodontidae | *Chaetodon trifascialis* |
| N | 1 | Chaetodontidae | *Heniochus chrysostomus* |
| N | 1 | Chaetodontidae | *Heniochus varius* |
| N | 1 | Labridae | *Labrichthys unilineatus* |
| N | 1 | Monacanthidae | *Oxycheilinus diaghrammus* |
| N | 1 | Monacanthidae | *Oxymonacanthus longirostris* |
| N | 2 | Labridae | *Labroides bicolor* |
| N | 2 | Labridae | *Labroides dimidiatus* |
| N | 3 | Balistidae | *Rhineacanthus aculeatus* |
| N | 3 | Holocentridae | *Sargocentron spiniferum* |
| N | 3 | Labridae | *Epibulus insidiator* |
| N | 3 | Lethrinidae | *Letrhinus rubrioperculatus* |
| N | 3 | Lutjanidae | *Lutjanus fulviflamma* |
| N | 3 | Mullidae | *Parupeneus bifasciatus* |
| N | 3 | Mullidae | *Parupeneus cyclostomus* |
| N | 3 | Mullidae | *Parupeneus multifasciatus* |
| N | 3 | Nemipteridae | *Scolopsis bilineata* |
| N | 3 | Pinguipedidae | *Papapercis hexophtalma* |
| N | 3 | Apogonidae | *Apogon* spp. |
| N | 3 | Blenniidae | *Atrosalarias fuscus* |
| N | 3 | Blenniidae | *Meiacanthus oualensis* |
| N | 3 | Haemulidae | *Plectorhinchus gibbosus* |
| N | 3 | Holocentridae | *Myripristis* sp. |
| N | 3 | Holocentridae | *Neoniphon* sp. |
| N | 3 | Holocentridae | *Neoniphon argenteus* |
| N | 3 | Holocentridae | *Sargocentrum ittodai* |
| N | 3 | Holocentridae | *Sargocentrum* sp. |
| N | 3 | Labridae | *Cheilinus chlorurus* |
| N | 3 | Labridae | *Cheilio inermis* |
| N | 3 | Labridae | *Coris aygula* |
| N | 3 | Labridae | *Coris gaimard* |
| N | 3 | Labridae | *Gomphosus varius* |
| N | 3 | Labridae | *Halichoeres hortulanus* |
| N | 3 | Labridae | *Halichoeres melannurus* |
| N | 3 | Labridae | *Hemigymnus melapterus* |
| N | 3 | Labridae | *Novaculichthys taeniourus* |
| N | 3 | Labridae | *Stethojulis bananensis* |
| N | 3 | Labridae | *Thalassoma hardwicke* |
| N | 3 | Labridae | *Valenciennea longipinnis* |
| N | 3 | Labridae | *Valenciennea strigata* |
| N | 3 | Lethrinidae | *Lethrinus harak* |
| N | 3 | Lethrinidae | *Monotaxis grandoculis* |
| N | 3 | Lethrinidae | *Monotaxis heterodon* |
| N | 3 | Mullidae | *Mulloidichthys flavolineatus* |
| N | 3 | Mullidae | *Mulloidichthys vanicolensis* |
| N | 3 | Mullidae | *Parapeneus barberinus* |
| N | 3 | Mullidae | *Parupeneus trifasciatus* |
| N | 4 | Apogonidae | *Apogon exostigma* |
| N | 4 | Apogonidae | *Apogon nigrofasciatus* |
| N | 4 | Chaetodontidae | *Chaetodon raffflesi* |
| N | 4 | Labridae | *Halichoeres trimaculatus* |
| N | 4 | Labridae | *Thalassoma jansenii* |
| N | 4 | Labridae | *Thalassoma lunare* |
| N | 4 | Zanclidae | *Zanclus cornutus* |
| N | 5 | Chaetodontidae | *Chaetodon auriga* |
| N | 5 | Chaetodontidae | *Chaetodon bennetti* |
| N | 5 | Chaetodontidae | *Chaetodon citrinellus* |
| N | 5 | Chaetodontidae | *Chaetodon ephipium* |
| N | 5 | Chaetodontidae | *Chaetodon lineolatus* |
| N | 5 | Chaetodontidae | *Chaetodon lunula* |
| N | 5 | Chaetodontidae | *Chaetodon ulietensis* |
| N | 5 | Chaetodontidae | *Chaetodon unimaculatus* |
| N | 5 | Chaetodontidae | *Chaetodon vagabundus* |
| N | 5 | Gobiidae | *Amblygobius phalaena* |
| N | 5 | Gobiidae | *Corythoichthys* sp. |
| N | 5 | Gobiidae | *Ctenogobips* sp. |
| N | 6 | Carangidae | *Caranx lugubris* |
| N | 6 | Carcharhinidae | *Carcharhinus melanopterus* |
| N | 6 | Fistulariidae | *Fistularia commersonii* |
| N | 6 | Labridae | *Plagiotremus rhinorhynchos* |
| N | 6 | Lutjanidae | *Lutjanus bohar* |
| N | 6 | Lutjanidae | *Lutjanus fulvus* |
| N | 6 | Muraenidae | *Gymnothorax* sp. |
| N | 6 | Epinephelidae | *Epinephelus* spp. |
| N | 6 | Labridae | *Plagiotremus laudandus* |

*Main categories: H – Herbivores , N – Non-herbivores.

**Sub categories as follows: within Herbivores 1 – Browsers, 2 – Grazers, 3 – Scraping parrotfishes, 4 – Excavating parrotfishes; within Non-herbivores 1 – Corallivores, 2 Cleaners, 3 – Mobile Invertebrate Feeders, 4 – Sessile invertebrate feeders, 5 – Omnivores, 6 – Piscivores.
