# Supplementary material for: Small Marine Protected Areas in Fiji Provide Refuge for Reef Fish Assemblages, Feeding Groups, and Corals
Source: PLoS One. 2017 Jan 25;12(1):e0170638. doi: 10.1371/journal.pone.0170638 (PMC5266309; doi:10.1371/journal.pone.0170638)
Supplement: S2 Table — Results of the models for the observed density and biomass of herbivorous reef fishes (per 120 m2). Parameter estimates (posterior mean), with 95% credible interval (CI) and effective sample size (ESS), for each level and interactions between levels of fixed factors (and variance associated with random factors). Effect sizes of the interaction site:status are relative to benchmark levels (non-MPAs to MPAs of each site). Text in bold highlights the effects deemed significant according to the 95% CI. (DOCX) [file pone.0170638.s004.docx]

**S2 Table. Mixed models herbivorous fish.** Results f the models for the observed density and biomass of herbivorous reef fishes (per 120 m^2^). Parameter estimates (posterior mean), with 95% credible interval (CI) and effective sample size (ESS), for each level and interactions between levels of fixed factors (and variance associated with random factors). Effect sizes of the interaction site:status are relative to benchmark levels (non-MPAs to MPAs of each site). Text in bold highlight the effects deemed significant according to the 95% CI.

|  | Density | | | | | Biomass | | | | | |
| --- | --- | --- | --- | --- | --- | --- | --- | --- | --- | --- | --- |
| Effect | Estimate | 95% CI | | ESS | | Estimate | 95% CI | | | ESS | |
| Feeding category: site: status |  |  |  | |  |  | |  |  | |  |
| Browsers |  |  |  | |  |  | |  |  | |  |
| **Votua** | **-1.51** | **-2.84** | **-0.08** | | **3098.91** | -3.22 | | -187.23 | 184.06 | | 5000 |
| Vatu-o-lalai | 0.68 | -0.52 | 1.89 | | 4259.38 | -0.53 | | -197.51 | 196.57 | | 5000 |
| **Namada** | **1.66** | **0.42** | **2.98** | | **4009.65** | 10.06 | | -205.33 | 231.26 | | 6000.58 |
| Grazers |  |  |  | |  |  | |  |  | |  |
| **Votua** | **-1.99** | **-2.53** | **-1.43** | | **5000** | **-269.27** | | **-439.82** | **-96.79** | | **5000** |
| **Vatu-o-lalai** | **-1.05** | **-1.48** | **-0.6** | | **5000** | **-390.41** | | **-568.51** | **-209.52** | | **5000** |
| **Namada** | **-0.95** | **-1.37** | **-0.49** | | **4512.92** | **-516.24** | | **-714.37** | **-311.2** | | **5000** |
| Scraping parrotfishes |  |  |  | |  |  | |  |  | |  |
| **Votua** | **-1.24** | **-1.67** | **-0.81** | | **4201.88** | **-449.94** | | **-621.4** | **-280.99** | | **5000** |
| Vatu-o-lalai | -0.27 | -0.7 | 0.12 | | 5000 | -193.66 | | -375.24 | 2.89 | | 5000 |
| Namada | -0.18 | -0.59 | 0.22 | | 5000 | -0.28 | | -194.57 | 180.82 | | 5000 |
| Excavating parrotfishes |  |  |  | |  |  | |  |  | |  |
| **Votua** | **-2.15** | **-2.76** | **-1.49** | | **5037.72** | **-259.32** | | **-437.75** | **-86.03** | | **4687.3** |
| **Vatu-o-lalai** | **-0.66** | **-1.17** | **-0.15** | | **5000** | -124.37 | | -322.31 | 54.69 | | 5000 |
| **Namada** | **-0.5** | **-0.97** | **-0.03** | | **4776.7** | -77.65 | | -257.55 | 109.86 | | 5000 |
| Random |  |  |  | |  |  | |  |  | |  |
| Votua:transects | 0.004 | 0 | 0.012 | | 5000 | 862.682 | | 0 | 3280.169 | | 4613.664 |
| Vatu-o-lalai:transects | 0.004 | 0 | 0.013 | | 5000 | 1497.666 | | 0 | 4844.372 | | 5000 |
| Namada:transects | 0.003 | 0 | 0.01 | | 5000 | 124540.458 | | 110938.142 | 138513.376 | | 5000 |
| Sampling day | 0.022 | 0 | 0.078 | | 4721.191 | -3.22 | | -187.23 | 184.06 | | 5000 |
| Residual | 0.561 | 0.456 | 0.671 | | 5730.425 | -0.53 | | -197.51 | 196.57 | | 5000 |
