# Supplementary material for: Small Marine Protected Areas in Fiji Provide Refuge for Reef Fish Assemblages, Feeding Groups, and Corals
Source: PLoS One. 2017 Jan 25;12(1):e0170638. doi: 10.1371/journal.pone.0170638 (PMC5266309; doi:10.1371/journal.pone.0170638)
Supplement: S3 Table — Results of the models for the observed density and biomass of non-herbivorous reef fishes (per 120 m2). Parameter estimates (posterior mean), with 95% credible interval (CI) and effective sample size (ESS), for each level and interactions between levels of fixed factors (and variance associated with random factors). Effect sizes of the interaction site:status are relative to benchmark levels (non-MPAs to MPAs of each site). Text in bold highlights the effects deemed significant according to the 95% CI. (DOCX) [file pone.0170638.s005.docx]

**S3 Table. Mixed models carnivorous fish.** Results f the models for the observed density and biomass of carnivorous reef fishes (per 120 m^2^). Parameter estimates (posterior mean), with 95% credible interval (CI) and effective sample size (ESS), for each level and interactions between levels of fixed factors (and variance associated with random factors). Effect sizes of the interaction site:status are relative to benchmark levels (non-MPAs to MPAs of each site). Text in bold highlight the effects deemed significant according to the 95% CI.

|  | Density | | | | | Biomass | | | | | |
| --- | --- | --- | --- | --- | --- | --- | --- | --- | --- | --- | --- |
| Effect | Estimate | 95% CI | | ESS | | Estimate | 95% CI | | | ESS | |
| Feeding category: site: status |  |  |  | |  |  | |  |  | |  |
| Cleaners |  |  |  | |  |  | |  |  | |  |
| Votua | ***** | ***** | ***** | | ***** | 1.89 | | -67.23 | 71.94 | | 5000 |
| Vatu-o-lalai | **-2.67** | **-5.47** | **-0.46** | | **1094.48** | -0.44 | | -72.11 | 71.22 | | 5000 |
| Namada | **-2** | **-3.79** | **-0.32** | | **2654.42** | 1.33 | | -73.3 | 76.47 | | 5000 |
| Corallivores |  |  |  | |  |  | |  |  | |  |
| Votua | **-1.56** | **-2.42** | **-0.76** | | **5000** | -43.9 | | -106.8 | 15.57 | | 5504.15 |
| Vatu-o-lalai | **-2.2** | **-2.99** | **-1.44** | | **5000** | **-108.19** | | **-183.9** | **-33.1** | | **5574.36** |
| Namada | **-1.6** | **-2.38** | **-0.83** | | **5367.76** | -50 | | -111.91 | 15.56 | | 5220.89 |
| Mobile invertebrate feeders |  |  |  | |  |  | |  |  | |  |
| Votua | 0.03 | -0.35 | 0.43 | | 5000 | 54.06 | | -4.32 | 115.64 | | 5000 |
| Vatu-o-lalai | **-0.59** | **-1.01** | **-0.16** | | **5000** | **-161.73** | | **-227.27** | **-91.76** | | **4721.49** |
| Namada | **-0.58** | **-1.01** | **-0.19** | | **5000** | **-172.87** | | **-238.66** | **-107.16** | | **5000** |
| Sessile invertebrate feeders |  |  |  | |  |  | |  |  | |  |
| Votua | **-0.58** | **-1.05** | **-0.05** | | **5238.25** | -21.96 | | -86.02 | 40.99 | | 5000 |
| Vatu-o-lalai | 0.36 | -0.18 | 0.87 | | 5927.64 | -0.72 | | -63.08 | 63.8 | | 4425.54 |
| Namada | 0.32 | -0.16 | 0.76 | | 5000 | -7.63 | | -71.64 | 53.55 | | 5000 |
| Omnivores |  |  |  | |  |  | |  |  | |  |
| Votua | -0.03 | -0.56 | 0.53 | | 5000 | -29.74 | | -88.88 | 31.6 | | 5319.94 |
| Vatu-o-lalai | -0.4 | -0.93 | 0.12 | | 5000 | -59.77 | | -125.31 | 11.44 | | 5000 |
| Namada | -0.43 | -0.96 | 0.11 | | 5000 | **-76.37** | | **-140.91** | **-13.56** | | **5000** |
| Piscivores |  |  |  | |  |  | |  |  | |  |
| Votua | **-1.28** | **-1.97** | **-0.63** | | **5000** | -43.82 | | -100.77 | 20.54 | | 5348.05 |
| Vatu-o-lalai | **-1.5** | **-2.07** | **-0.91** | | **5000** | **-107.71** | | **-176.74** | **-40.07** | | **4758.56** |
| Namada | **-1.21** | **-1.77** | **-0.56** | | **5000** | **-75** | | **-146.43** | **-7.61** | | **5000** |
| Random |  |  |  | |  |  | |  |  | |  |
| Votua:transects | 0.005 | 0 | 0.019 | | 5000 |  | |  |  | |  |
| Vatu-o-lalai:transects | 0.021 | 0.001 | 0.085 | | 5000 |  | |  |  | |  |
| Namada:transects | 0.005 | 0 | 0.015 | | 5000 |  | |  |  | |  |
| Transects |  |  |  | |  | 324.709 | | 0 | 1030.497 | | 5235.131 |
| Sampling day | 0.565 | 0.466 | 0.68 | | 5000 | 255.014 | | 0 | 637.886 | | 5000 |
| Residual | 0.021 | 0.001 | 0.085 | | 5000 | 15260.365 | | 13924.897 | 16641.904 | | 5330.659 |

*this interaction could not be estimated due to small sample size
