# Supplementary material for: Small Marine Protected Areas in Fiji Provide Refuge for Reef Fish Assemblages, Feeding Groups, and Corals
Source: PLoS One. 2017 Jan 25;12(1):e0170638. doi: 10.1371/journal.pone.0170638 (PMC5266309; doi:10.1371/journal.pone.0170638)
Supplement: S4 Table — Results of models for the number of individuals, biomass (kg) and diversity of species (Shannon diversity index) of fishes in feeding groups in MPAs and adjacent non-MPAs at three village sites (Votua, Vatu-o-lalai, and Namada) along the Coral Coast of Fiji. Parameter estimates (posterior mean), with 95% credible interval (CI) and effective sample size (ESS), for each level and interactions between levels of fixed factors (and variance associated with random factors). Effect sizes of the interaction site:status are relative to benchmark levels (MPAs of each site). Text in bold highlights the effects deemed significant according to the 95% CI. (DOCX) [file pone.0170638.s006.docx]

**Table S4. Fish groups.** Results of models for the number of individuals, biomass (kg) and diversity of species (Shannon diversity index) of fishes in feeding groups in MPAs and adjacent non-MPAs at three village sites (Votua, Vatu-o-lalai, and Namada) along the Coral Coast of Fiji. Parameter estimates (posterior mean), with 95% credible interval (CI) and effective sample size (ESS), for each level and interactions between levels of fixed factors (and variance associated with random factors). Effect sizes of the interaction site:status are relative to benchmark levels (MPAs of each site). Text in bold highlight the effects deemed significant according to the 95% CI.

|  | Number of individuals | | | | Fish biomass (kg) | | | | Species diversity | | | |
| --- | --- | --- | --- | --- | --- | --- | --- | --- | --- | --- | --- | --- |
| Effect | Estimate | 95% CI | | ESS | Estimate | 95% CI | | ESS | Estimate | 95% CI | | ESS |
| Site: status |  |  |  |  |  |  |  |  |  |  |  |  |
| Votua | 0.16 | -0.05 | 0.33 | 5000 | **-51.32** | **-71.36** | **-31.52** | **5220.21** | **-0.22** | **-0.3** | **-0.13** | **5000** |
| Vatu-o-lalai | 0.04 | -0.14 | 0.22 | 5000 | **-55.88** | **-73.61** | **-36.07** | **5388.95** | **-0.1** | **-0.18** | **-0.02** | **5000** |
| Namada | -0.05 | -0.26 | 0.15 | 4713.72 | **-64.2** | **-85.35** | **-44.3** | **5000** | -0.07 | -0.15 | 0.02 | 5000 |
| Random |  |  |  |  |  |  |  |  |  |  |  |  |
| Votua: transects | 0.004 | 0 | 0.01 | 5000 | 0.004 | 0 | 0.012 | 3807.11 |  |  |  |  |
| Vatu-o-lalai: transects | 0.003 | 0 | 0.007 | 5000 | 0.004 | 0 | 0.011 | 5000 | 0.002 | 0 | 0.005 | 4664.19 |
| Namada: transects | 0.05 | 0.001 | 0.129 | 5000 | 0.004 | 0 | 0.012 | 3941.85 | 0.003 | 0 | 0.008 | 5000 |
| Residual | 0.44 | 0.38 | 0.508 | 5000 | 6337.33 | 5737.68 | 6973.08 | 5075.10 | 0.003 | 0 | 0.007 | 5000 |
