# Supplementary material for: Small Marine Protected Areas in Fiji Provide Refuge for Reef Fish Assemblages, Feeding Groups, and Corals
Source: PLoS One. 2017 Jan 25;12(1):e0170638. doi: 10.1371/journal.pone.0170638 (PMC5266309; doi:10.1371/journal.pone.0170638)
Supplement: S5 Table — Statistical differences in percent cover (arcsine-transformed data) of scleractinian corals, macroalgae, epilithic algal matrix (EAM) and “others” in MPAs and adjacent non-MPAs at the villages of Votua, Vatu-o-lalai, and Namada along the Coral Coast of Fiji. Significant p-values marked in bold (p < 0.05); highly significant p-values (< 0.0001) marked with **. (DOCX) [file pone.0170638.s007.docx]

**S5 Table. ANOVA of benthic cover.** Statistical differences in percent cover (arcsine-transformed data) of scleractinian corals, macroalgae, epilithic algal matrix (EAM) and “others” in MPAs and adjacent non-MPAs at the villages of Votua, Vatu-o-lalai, and Namada along the Coral Coast of Fiji. Significant p-values marked in **bold** (p < 0.05).

|  | **SS** | ***df*** | **MS** | **F** | **p** |
| --- | --- | --- | --- | --- | --- |
| **Scleratinian corals** | | | | | |
| Site | 0.25 | 2 | 0.12 | 10.09 | **<0.001** |
| Status | 2.26 | 1 | 2.26 | 182.22 | **<0.001** |
| Year | 0.00 | 1 | 0.00 | 0.02 | 0.89 |
| Site.status | 0.02 | 2 | 0.01 | 0.91 | 0.40 |
| Site.year | 0.00 | 2 | 0.00 | 0.18 | 0.84 |
| Status.year | 0.00 | 1 | 0.00 | 0.00 | 0.98 |
| Site.status.year | 0.00 | 2 | 0.00 | 0.01 | 0.99 |
| Residuals | 3.24 | 261 | 0.01 |  |  |
| **Macroalgae** |  |  |  |  |  |
| Site | 1.04 | 2 | 0.52 | 24.42 | **<0.001** |
| Status | 4.67 | 1 | 4.67 | 220.19 | **<0.001** |
| Year | 0.00 | 1 | 0.00 | 0.02 | 0.89 |
| Site.status | 0.12 | 2 | 0.06 | 2.82 | 0.06 |
| Site.year | 0.04 | 2 | 0.02 | 1.01 | 0.37 |
| Status.year | 0.01 | 1 | 0.01 | 0.74 | 0.39 |
| Site.status.year | 0.01 | 2 | 0.00 | 0.34 | 0.71 |
| Residuals | 5.54 | 261 |  |  |  |
| **Epilithic algal matrix** | | | | | |
| Site | 1.37 | 2 | 0.68 | 30.18 | **<0.001** |
| Status | 0.00 | 1 | 0.00 | 0.00 | 0.99 |
| Year | 0.00 | 1 | 0.00 | 0.24 | 0.62 |
| Site.status | 0.24 | 2 | 0.05 | 5.35 | **0.006** |
| Site.year | 0.02 | 2 | 0.12 | 0.36 | 0.70 |
| Status.year | 0.03 | 1 | 0.03 | 1.26 | 0.26 |
| Site.status.year | 0.04 | 2 | 0.02 | 0.90 | 0.41 |
| Residuals | 5.93 | 261 |  |  |  |
| **Others** |  |  |  |  |  |
| Site | 0.63 | 2 | 0.31 | 14.81 | **<0.001** |
| Status | 0.10 | 1 | 0.10 | 4.70 | **0.03** |
| Year | 0.00 | 1 | 0.00 | 0.12 | 0.73 |
| Site.status | 0.13 | 2 | 0.06 | 2.97 | 0.05 |
| Site.year | 0.06 | 2 | 0.03 | 1.24 | 0.29 |
| Status.year | 0.01 | 1 | 0.01 | 0.44 | 0.51 |
| Site.status.year | 0.02 | 2 | 0.01 | 0.57 | 0.57 |
| Residuals | 5.56 | 261 |  |  |  |
