# Supplementary material for: Small Marine Protected Areas in Fiji Provide Refuge for Reef Fish Assemblages, Feeding Groups, and Corals
Source: PLoS One. 2017 Jan 25;12(1):e0170638. doi: 10.1371/journal.pone.0170638 (PMC5266309; doi:10.1371/journal.pone.0170638)
Supplement: S6 Table — Comparison of percentage cover of scleractinian corals and macroalgae in MPAs and adjacent non-MPAs at three village sites (Votua, Vatu-o-lalai, and Namada) along the Coral Coast of Fiji. P-values (Holm-Sídák adjusted; df = 261) refer to planned comparisons following ANOVA. Significant p-values marked in bold (p < 0.05); highly significant p-values (< 0.0001) marked with **. (DOCX) [file pone.0170638.s008.docx]

**S6 Table. MPA vs non-MPA benthic cover.** Comparison of percentage cover of scleractinian corals and macroalgae in MPAs and adjacent non-MPAs at three village sites (Votua, Vatu-o-lalai, and Namada) along the Coral Coast of Fiji. P-values (Holm-Sídák adjusted; *df* = 261) refer to planned comparisons following ANOVA. Significant p-values marked in **bold** (p < 0.05).

|  | Percentage cover, mean ± SE (n) | |  |  |
| --- | --- | --- | --- | --- |
| Village | MPA | non-MPA | t-ratio | p-adj |
| **Scleractinian corals** | | | | |
| Votua | 22.53 ± 1.59 (48) | 8.53 ± 0.93 (39) | 8.55 | **<0.001** |
| Vatu-o-lalai | 21.50 ± 1.34 (44) | 10.19 ± 0.78 (50) | 6.85 | **<0.001** |
| Namada | 17.56 ± 1.11 (41) | 6.25 ± 0.52 (51) | 7.82 | **<0.001** |
| **Macroalgae** |  |  |  |  |
| Votua | 6.26 ± 1.13 | 24.72 ± 2.57 | -8.81 | **<0.001** |
| Vatu-o-lalai | 1.84 ± 0.74 | 10.48 ± 1.20 | -6.89 | **<0.001** |
| Namada | 0.68 ± 0.13 | 014.86 ± 1.41 | -9.93 | **<0.001** |
